# The potential and challenges of targeting *MTAP*-negative cancers beyond synthetic lethality

**DOI:** 10.3389/fonc.2023.1264785

**Published:** 2023-09-19

**Authors:** Chandler Bray, Cristina Balcells, Iain A. McNeish, Hector C. Keun

**Affiliations:** ^1^ Cancer Metabolism & Systems Toxicology Group, Division of Cancer, Department of Surgery & Cancer, Imperial College London, London, United Kingdom; ^2^ Ovarian Cancer Action Research Centre, Department of Surgery and Cancer, Imperial College London, London, United Kingdom

**Keywords:** MTAP, MAT2A, PRMT5, methionine, metabolism, synthetic lethality

## Abstract

Approximately 15% of cancers exhibit loss of the chromosomal locus 9p21.3 – the genomic location of the tumour suppressor gene *CDKN2A* and the methionine salvage gene *methylthioadenosine phosphorylase* (*MTAP*). A loss of MTAP increases the pool of its substrate methylthioadenosine (MTA), which binds to and inhibits activity of protein arginine methyltransferase 5 (PRMT5). PRMT5 utilises the universal methyl donor S-adenosylmethionine (SAM) to methylate arginine residues of protein substrates and regulate their activity, notably histones to regulate transcription. Recently, targeting PRMT5, or MAT2A that impacts PRMT5 activity by producing SAM, has shown promise as a therapeutic strategy in oncology, generating synthetic lethality in *MTAP*-negative cancers. However, clinical development of PRMT5 and MAT2A inhibitors has been challenging and highlights the need for further understanding of the downstream mediators of drug effects. Here, we discuss the rationale and methods for targeting the MAT2A/PRMT5 axis for cancer therapy. We evaluate the current limitations in our understanding of the mechanism of MAT2A/PRMT5 inhibitors and identify the challenges that must be addressed to maximise the potential of these drugs. In addition, we review the current literature defining downstream effectors of PRMT5 activity that could determine sensitivity to MAT2A/PRMT5 inhibition and therefore present a rationale for novel combination therapies that may not rely on synthetic lethality with *MTAP* loss.

## 1 Introduction

### 1.1 *MTAP* deletion creates therapeutic vulnerabilities in tumours

Gain-of-function or activating genetic alterations that occur in many cancers have proven useful as precision therapy targets. However, loss-of-function alterations must be targeted indirectly. To utilise these alterations for therapy, there must be a thorough understanding of the altered processes associated with the loss of gene products and any cancer-specific susceptibilities that may arise. Large genomic deletions that occur in cancers can lead to a growth or survival advantage by loss of tumour suppressor function. However, co-deletion of other genetic material in close physical proximity (“passenger deletions”) may create new “synthetically lethal” or “collateral” vulnerabilities to therapy (1, 2). The chromosome 9p21.3 region is deleted in approximately 15% of cancers (3, 4) and contains the tumour suppressor gene *cyclin-dependent kinase inhibitor 2A (CDKN2A).* This gene encodes for the p14 (5) and p16 (6) proteins that stabilise p53 and block G1 progression respectively. Only 100 kb away from the *CDKN2A* locus resides a key gene in the methionine metabolism cycle, *5’-deoxy-5’-methylthioadenosine phosphorylase (MTAP)* (7)*. MTAP* is co-deleted with homozygous loss of *CDKN2A* in approximately 80-90% of cases of 9p21.3 homozygous deletion (8) and its loss is associated with poor prognosis (9–11). The co-occurrence of *CDKN2A/MTAP* deletion may explain early literature that observed *MTAP* loss in leukaemia (12, 13) and breast cancer (14).

**Figure 1:**
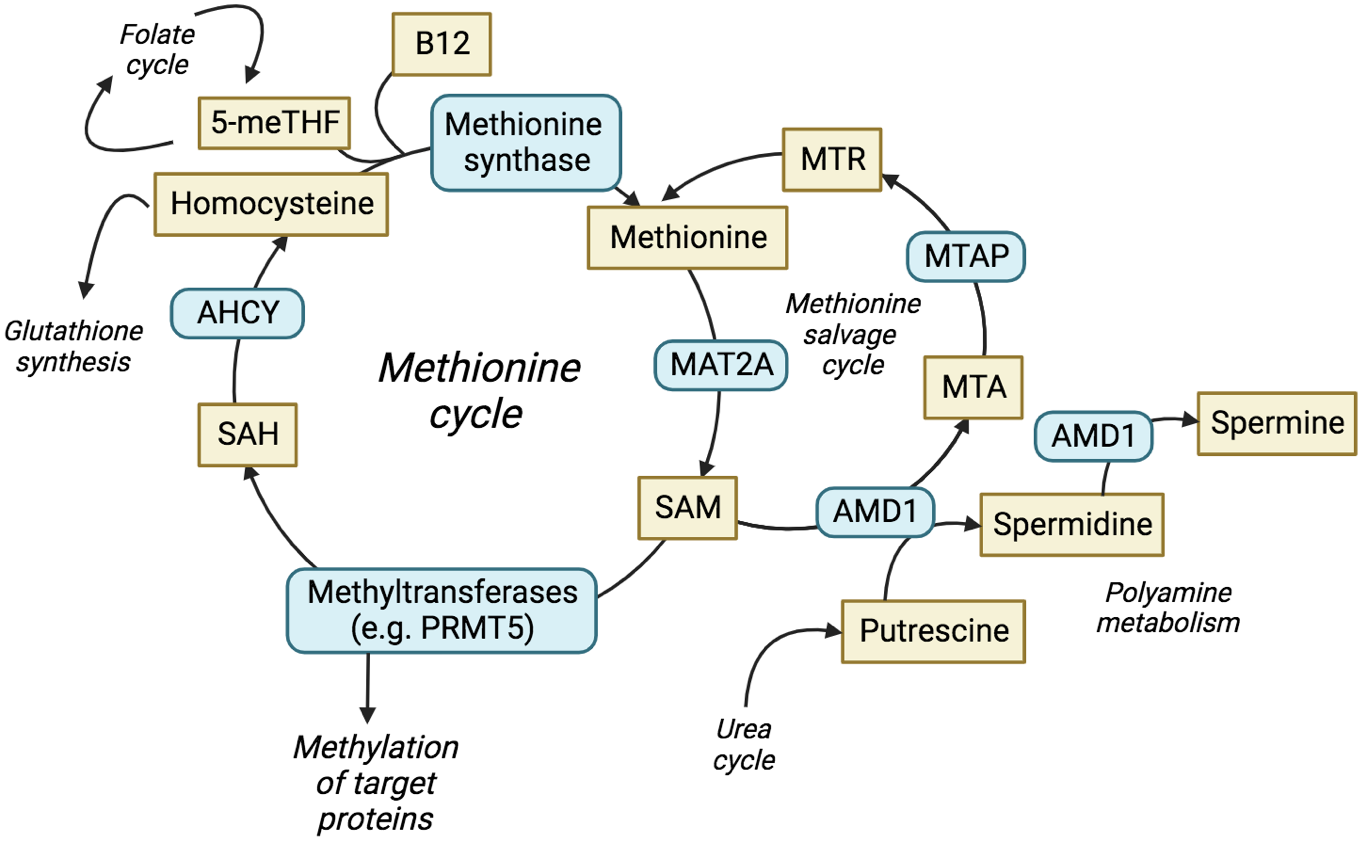
The methionine cycle and interrelated pathways. The production and utilisation of SAM is central to the methionine cycle and allows protein methylation by methyltransferases. The methionine salvage cycle recovers methionine through the conversion of MTA to methionine via the analogue MTR. ACHY, adenosylhomocysteine; AMD1, adenosylmethionine decarboxylase 1; ASA, arginonosuccinic acid; MTR, methylthioribose; ODC, ornithine decarboxylase; SAH, S-adenosyl-homocysteine; THF, tetrahydrofolate.

MTAP metabolises methylthioadenosine (MTA) in the methionine salvage cycle, regenerating methionine for further cycling (**Figure 1**) (15). Methionine is an essential amino acid and loss of MTAP increases cellular dependence upon exogenous methionine (16), with implications for nucleotide synthesis, folate metabolism, glutathione synthesis and the urea cycle. MTA is a product of the synthesis of the universal methyl donor, S-adenosylmethionine (SAM), and the polyamine synthesis pathway. MTA was shown to be secreted into culture medium by *MTAP*-negative leukaemia cells *in vitro* (17), whilst elevated levels of MTA and MTA secretion have frequently been observed in multiple cell lines derived from solid tumours with homozygous deletion of *MTAP* (18–20). MTAP is the only enzyme known to metabolise MTA, which highlights a lack of redundancy in this process and suggests that targeting methionine metabolism may be an effective therapy against *MTAP*-negative malignancies. Furthermore, loss of 9p21.3 has been shown to be a driver, or trunk event, that occurs early in cancer evolution (21). This suggests that *MTAP* deletion is present before additional branch mutations have occurred, i.e. before other adaptive processes can take place.

Multiple studies in 2016 undertook short hairpin RNA (shRNA) screens to identify genes that cause synthetic lethality with both endogenous and genetically engineered loss of *MTAP* (18–20). All provided evidence of a conditional dependence on protein arginine methyl transferase 5 (PRMT5), RIOK1, WDR77 (MEP50) and methionine adenosyltransferase II alpha (MAT2A). These proteins are involved in the methionine cycle or subsequent methylation reactions: MAT2A catalyses the direct production of SAM (22, 23); PRMT5 and its associated binding partners RIOK1 (24) and MEP50 (25, 26) utilise SAM as methyl donor to methylate specific protein targets.

In this review, we examine the current understanding of therapeutic vulnerabilities intrinsic to *MTAP*-negative tumours, focusing on MAT2A and PRMT5, which are receiving increasing attention in clinical development. We discuss the function of MAT2A and PRMT5, including binding partners, current methods of inhibition, downstream signalling and effect on metabolic pathways. We review the interplay between these proteins, and how therapeutic inhibition impacts growth, cell cycle, apoptosis or DNA damage response. Finally, we highlight the challenges that face the therapeutic targeting of the MAT2A/PRMT5 axis, the need for additional predictive biomarkers other than *MTAP* status, and how these biomarkers could predict rational combination therapies.

## 2 The structure and function of PRMT5

PRMT5 is a member of the family of protein arginine methyltransferases responsible for methylating specific arginine residues of a wide range of proteins and thus regulate protein activity. This includes histones that regulate chromatin structure and epigenetic regulation of gene expression (27). The nine members of the PRMT family can be split into four distinct types that distinguish their activity. Type I, II and III PRMTs catalyse the formation of monomethylarginine intermediates at the terminal guanidino nitrogen atom of arginine, which can be subsequently modified to produce asymmetric dimethylarginines (ADMA) by the type I PRMTs (e.g. PRMT1) or symmetric dimethylarginines (SDMA) by the type II PRMTs (e.g. PRMT5). Type IV PRMTs can also monomethylate arginine, but they do this at the internal guanidine nitrogen atom of arginine (28, 29).

**Figure 2:**
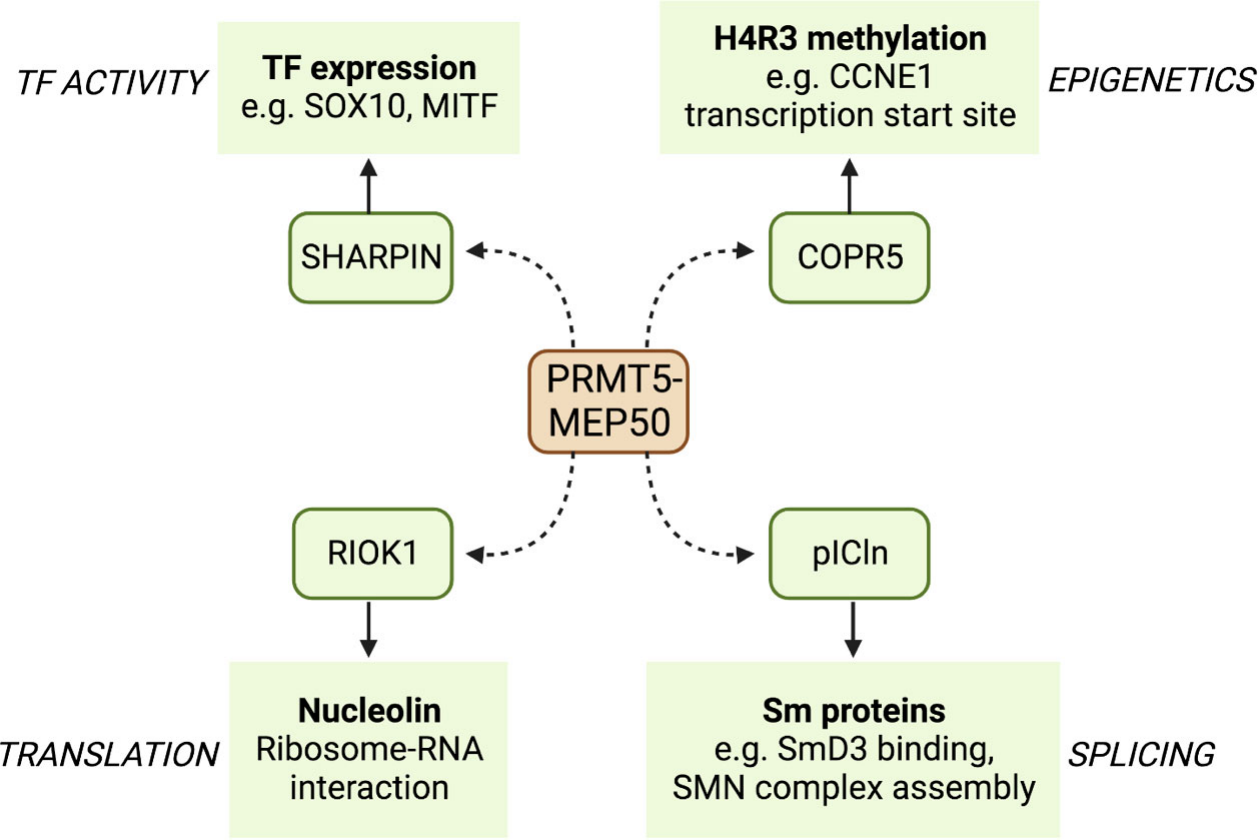
A selection of binding partners (SAPS) of PRMT5 and some of their targets. PRMT5 binds to the PBM of a number of SAPs that localise its arginine methylation activity to different sites. Here, we show four PRMT5 SAPs that act as adapters to specify binding to transcription factors such as SOX10 and MITF, translation regulating proteins such as nucleolin, splicing factors such as SmD3, and CDK regulators such as the cyclin CCNE1.

PRMT5 is the principal type II PRMT and functions mainly as a negative regulator of transcription (30). PRMT5 contains two distinct domains: a C-terminal catalytic domain that interacts with the methyl donor SAM and a N-terminal TIM barrel domain that allows interaction with binding partners such as MEP50 (26). PRMT5 binds to MEP50 to produce PRMT5-MEP50 heterodimers, which in turn form a tetramer complex; these interactions are essential for stimulating the activity of PRMT5. SAM is then utilised by the complex as a methyl donor to allow the addition of the methyl group to the target arginine. PRMT5 binds to substrate adaptor proteins (SAPs) that are required for PRMT5 targeting and subsequent methylation. All SAPs share the peptide sequence GQF[D/E]DA[D/E] known as the PRMT5 binding motif (PBM), which facilitates PRMT5 binding (31), and the specific SAP that binds to PRMT5 can localise its activity to different substrates (**Figure 2**). The PRMT5 complex has been shown to preferentially bind an arginine- and glycine-rich (GRG) domain in the target substrate proteins as the site of arginine methylation (32). More than 100 substrates have been identified that are methylated by PRMT5 to regulate their functions, including proteins that promote survival and tumorigenesis (32, 33).

## 3 PRMT5 activity and cancer

Many studies have identified that increased activity and upregulation of PRMT5 is a key regulator of cancer progression and marker for poor prognosis in multiple malignancies, including breast (34), gastric (35), glioblastoma (36), leukaemia (37), lung (38), lymphoma (39), ovarian (40), pancreatic (41) and prostate cancer (42). PRMT5 can act to promote tumorigenesis by methylating histone and non-histone proteins to regulate transcriptional and post-translational cell growth pathways, respectively (**Table 1**).

**Table 1:** PRMT5 targets.

PRMT5 target	Function	PRMT5 action on target	Cell line/cancer type	Citation
ALYREF	Pre-mRNA transport and splicing	Methylation of protein	THP-1 (AML)	(37)
AR	Tumour promoting gene	Increased expression via epigenetic regulation	LNCaP, C4-2 (Prostate cancer)	(42)
C-Myc	Regulate NF-κB pathway	Increased expression and stabilise protein	T24, 5637 (Bladder cancer), MUA PaCa-2, SW1990 (Pancreatic cancer)	(41, 43)
CANNTG	C-Myc-binding E-box element	Reduced expression of downstream genes via epigenetic regulation	BGC823 and SGC7901 (Gastric cancer)	(35)
CCNE1	G1/S transition via CDK2 regulation	Reduced expression via epigenetic regulation	U2OS	(44)
CCT4	Component of TRiC complex	Methylation of protein	THP-1 (AML)	(37)
CCT7	Component of TRiC complex	Methylation of protein	THP-1 (AML)	(37)
CD74	Component of MHC II molecule	Reduced expression via epigenetic regulation	Liver cancer	(45)
CDH1	Tumour suppressor gene	Reduced expression via epigenetic regulation	A549 (Lung cancer)	(46)
CIITA	Component of MHC II molecule	Reduced expression via epigenetic regulation	Liver cancer	(45)
CPSF6	3’ RNA cleavage and polyadenylation	Methylation of protein	THP-1 (AML)	(37)
E2F1	Transcription factor that can regulate apoptosis	Methylation and destabilisation of protein	U2OS	(47)
FA genes	Inter-strand crosslink (CIL)-induced DNA damage repair	Increased expression via epigenetic regulation	U251MG, T98G, U118MG (Glioblastoma)	(48)
FBW7	C-Myc regulator gene	Reduced expression via epigenetic regulation	PaCa-2, SW1990 (Pancreatic cancer)	(41)
FOXP1	Activates oestrogen receptor (ER)	Increased expression via epigenetic regulation	MCF7 (Breast cancer)	(34)
IFI16 (IFI204)	Regulator of STING/cGAS signalling pathway	Methylation of protein to control function	A375, WM115, B16 (Melanoma)	(49)
Mxi1	C-Myc agonist	Methylation of protein leading to degradation	H1299, A549, H460, H522, H358 (NSCLC)	(50)
NLRC5	Regulator of MHC I gene expression	Decreased expression of gene	B16, CCLE melanoma cell lines (Melanoma)	(49)
NM23	Tumour suppressor gene	Reduced expression via epigenetic regulation	NIH/3T3	(51)
p53	Promotes cell cycle arrest to allow DNA repair, regulates apoptosis	Methylation of protein to inactivate pro-apoptotic function	U2OS, HSPCs	(52, 53)
PNN	Part of exon junction complex (EJC)	Methylation of protein	THP-1 (AML)	(37)
RPS10	Component of the 40S ribosomal subunit	Methylation of protein	THP-1 (AML)	(37)
RUVBL1	Component of TIP60 complex for directing DNA damage repair towards the HR pathway	Methylation of protein to allow complex formation	HeLa (Cervical cancer)	(54)
SFPQ	Early splicing factor	Methylation of protein	THP-1 (AML)	(37)
Sm proteins	Formation of the spliceosome	Methylation required for smRNP biogenesis	HeLa (Cervical cancer)	(55)
SNAIL1	Epithelial-mesenchymal transition (EMT) and metastatic activator factor	Increased expression via epigenetic regulation	A549 (Lung cancer)	(46)
SNRPB	Component of SMN-Sm complex	Methylation of protein	THP-1 (AML)	(37)
SPDEF	Tumour suppressor gene	Reduced expression via epigenetic regulation	A549 (Lung cancer)	(46)
SRSF1	Prevents exon skipping	Methylation of protein	THP-1 (AML)	(37)
ST7	Tumour suppressor gene	Reduced expression via epigenetic regulation	NIH/3T3	(51)
SUPT5H	mRNA processing, transcription and elongation of RNAP II	Methylation of protein	THP-1 (AML)	(37)
VIM	Epithelial-mesenchymal transition (EMT) and metastatic activator factor	Increased expression via epigenetic regulation	A549 (Lung cancer)	(46)
WDR33	mRNA polyadenylation	Methylation of protein	THP-1 (AML)	(37)
ZNF326	Subunit of DBIRD complex that regulates alternative splicing	Methylation requires for accurate splicing	MDA-MB-231 (Breast cancer)	(56)

### 3.1 Epigenetic control of tumour regulating genes by PRMT5

Overexpression of PRMT5 has important consequences for the epigenetic landscape of cancer across different cancer types. An early study identified PRMT5 as a binding partner of hSWI/SNF complexes that cooperatively target tumour suppressor genes *ST7* and *NM23* to inhibit their transcription (51). Transcriptome profiling of PRMT5/MEP50 shRNA knockdown lung cancer models identified differential expression of components of the TGFβ pathway, suggesting that PRMT5 may be important for the TGFβ response and subsequent cancer metastasis (46). Knockdown of PRMT5 and MEP50 showed a reduction of activating epigenetic methylation markers (H3R2me1 and H3R4me3) at the promoters of *SNAIL1* and *VIM*, both key epithelial-mesenchymal transition (EMT) and metastasis activator genes. In the same conditions, a reduction of repressive marks (H4R3me2) at the tumour suppressor genes *SPDEF* and *CDH1* was observed (46). Co-operator of PRMT5 (COPR5), a SAP, is essential for PRMT5 binding and H4R3 methylation at transcription starts sites of genes such as *CCNE1* (44). In prostate cancer cells, the *androgen receptor (AR)* promoter was shown to be an epigenetic target of PRMT5, and knockdown of PRMT5 caused a reduction in H4R3me2 marks at the *AR* promoter and a subsequent reduction in *AR* expression (42). In breast cancer stem cells, it was shown that PRMT5 functions to methylate H3R2, allowing SET1 binding and H3K4 trimethylation at the *FOXP1* promoter to activate *FOXP1* transcription both *in vitro* and *in vivo*. The expressed FOXP1 protein promoted breast cancer cell proliferation by activating oestrogen receptor (ER) signalling (34). PRMT5 was reported to deposit H4R3me2 marks at the c-Myc-binding E-box element (CANNTG), and that, in addition to c-Myc, PRMT5 binding results in the silencing of downstream genes. The genes affected include negative regulators of cell cycle, such as *PTEN, CDKN2C, CDKN1A, CDKN1C* and *p63* (35). PRMT5 has also been shown to epigenetically silence the promoter region (via an increase of H4R3me2 and H3K9me3 marks) of the c-Myc regulator gene *FBW7* (41). A reduction in PRMT5 activity also has a negative regulatory effect on the DNA damage repair Fanconi Anaemia (FA) family genes via reduced H3R2 monomethylation markers at FA gene promoters (48). PRMT5 was also suggested to regulate MHC II expression by histone methylation at the promoters of *CD74* and *CIITA*, therefore affecting how tumours present to the immune system (45). Hence, PRMT5 has shown specific epigenetic control of a range of cancer-relevant genes and promotes growth and progression.

### 3.2 Transcription factor regulation by PRMT5

p53 is a widely studied tumour suppressor protein that responds to DNA damage by arresting growth and inducing an apoptotic response (57). PRMT5 has been shown to associate with STRAP (DNA damage cofactor) and p53, leading to the subsequent methylation of p53, whilst low expression of PRMT5 led to p53-induced apoptosis (52). The presence and methyltransferase activity of PRMT5 was later shown to be sufficient to inactivate p53 in haematopoietic stem progenitor cells (HSPCs), inhibit apoptosis and increase self-renewal *in vitro* and *in vivo* (53). Thus, an increase in PRMT5 expression will positively impact cancer proliferation.

As with p53, the transcription factor E2F1 can promote apoptosis by activating pro-apoptotic genes. PRMT5 has been found to methylate and destabilise E2F1 (47) and short interfering RNA (siRNA) knockdown of PRMT5 caused an increase in E2F1 mRNA and protein levels in ovarian cancer cells, resulting in decreased growth rate and induction of apoptosis (40). E2F1 can act in a mutually exclusive pro- or anti-proliferative manner, the former when marked with the symmetric methylation of R111/R113 by PRMT5. Conversely, asymmetric methylation of R109 by PRMT1 induces apoptosis. PRMT5 methylation of E2F1 and PRMT1 knockdown were both linked to a decrease in mRNA levels of apoptosis associated proteins (APAF1, p73 and E2F-1). Moreover, symmetric arginine methylation of E2F1 via PRMT5 was read by proliferation-promoting protein p100-TSN, which increased the binding of p100-TSN to promoters of proliferation-inducing genes (cyclin E, Cdc6 and DHFR) (58). Knockdown or inhibition of E2F1 and PRMT5 in HCT116 cells was later shown to lead to reduced expression of cell migration and invasion genes (e.g. *cortactin/CTTN*) and consequently defects in these processes. Further, *PRMT5/E2F1* expression and *cortactin/CTTN* expression showed positive correlations across different types of cancer in the Cancer Genome Atlas datasets (TCGA) (59), suggesting that E2F1 and PRMT5 regulate the process of cell migration and invasion.

PRMT5 has been reported to promote c-Myc expression and consequently up-regulate the NF-κB pathway (43). Furthermore, PRMT5 has been shown to stabilise c-Myc in pancreatic cancer cells (41). PRMT5 also methylates the c-Myc agonist Mxi1 to promote β-Trcp ligase-directed degradation of Mxi1. Consequently, inhibition of PRMT5 achieved radiosensitivity in non-small cell lung cancer (NSCLC) (50). These results highlight an important and widespread role of PRMT5 in promoting the oncogenic mechanisms of cancer. Reduction or inhibition of PRMT5 could therefore be an approach for treating cancer.

### 3.3 The role of PRMT5 in splicing and DNA damage repair

Seven small nuclear ribonucleoproteins (snRNPs) formed of Sm proteins and small nuclear RNA assemble to form the spliceosome (60). The spliceosome requires the snRNP assembly factor SMN to accurately assemble and bind at sites that require splicing (61). SMN binds the dimethylated arginine/glycine (GRG) domains of Sm proteins to allow for accurate recognition (62). Sm dimethylation is attributed to the 20S methyltransferase complex, or methylosome, comprising PRMT5, MEP50 and the SAP pICln (63). The methylosome (specifically PRMT5) acts to add SDMA modifications to Sm proteins that are required for snRNP biogenesis and the resulting process of splicing *in vivo* (55). Post-translational dimethylation of the splicing-associated protein ZNF326 was reduced upon PRMT5 inhibition by causing inclusion of AT rich introns in target genes, which phenocopied the loss of ZNF326 protein (56, 64). Deletion of *PRMT5* has been shown to cause perturbed splicing leading to reduced canonical, and increased alternative, splicing specifically in pre-mRNAs with a weak 5’ donor site (65). This study highlighted alternatively spliced *Mdm4* mRNA as a recurrent product of *PRMT5* deletion. *Mdm4* encodes for a key activator of p53 and alternative splicing leads to increased activation of the p53 transcription process and indicates a response to PRMT5 inhibitors (66).

**Figure 3:**
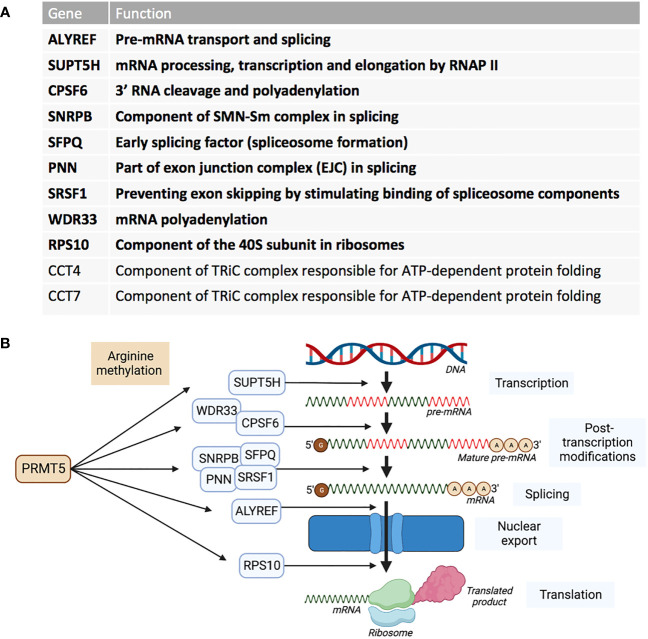
PRMT5 substrates associated with AML proliferation outlined by Radzisheuskaya et al., 2019. **(A)** The eleven PRMT5 substrate genes identified as responsible for proliferation in AML and their functions. The genes in bold represent the nine genes that are involved in RNA metabolism and splicing. **(B)** The nine RNA metabolism and splicing proteins that are methylated by PRMT5 and whereabouts they act in the process of RNA metabolism and splicing.

RNA sequencing of cells in which PRMT5 has been pharmacologically inhibited, demonstrated that a reduction in activity of PRMT5 caused an increase in detained introns (67). A detained intron (DI) describes the presence of a post-transcriptional intron in pre-mRNA that results in the transcript being detained within the nucleus (68). DI-containing transcripts are then either processed further by post-transcriptional splicing or degraded – leading to an overall reduction in the translated protein product (69). These types of alternative splicing upon PRMT5 downregulation in breast cancer cell line MDA-MB-231 were found to be enriched for transcription products associated with RNA processing such as splicing genes- *HNRNPC, HNRNPH1, RBM5, RBM23, RBM39* and *U2AF1* (56). In glioblastoma, PRMT5 inhibition globally increased abnormal splicing events, while mis-spliced transcripts were enriched in cell cycle progression pathways (70). Profiling the PRMT5 methylome identified 11 proteins that are essential in the proliferation of acute myeloid leukaemia (AML) cells (37). Nine of these PRMT5 substrates are regulators of RNA metabolism and splicing (**Figure 3**). AML cells were also shown to have increased DI-containing transcripts encoding the transcription factor ATF4 when PRMT5 was inhibited, decreasing levels of ATF4 and increasing oxidative stress and senescence (71). AML cells with genetic abnormalities in splicing gene *Srsf2* were preferentially killed over *Srsf2* WT cells when treated with PRMT5 inhibitors (72).

PRMT5 methylation has been identified as an important regulating process for the DNA damage repair pathways. Under normal conditions, the TIP60 (KAT5) complex acetylates H4K16, displaces the non-homologous end joining (NHEJ)-promoting 53BP1 protein and directs DNA damage repair towards the homologous recombination (HR) pathway (54, 73). By contrast, PRMT5 deficiency leads to alternative splicing of TIP60, impairing acetyltransferase activity (73). In addition, PRMT5 directly methylates the TIP60 complex cofactor protein RUVBL1 that is essential for accurate complex function (54). In a PRMT5-deficient environment, the TIP60 complex cannot function to promote HR and consequently error-prone NHEJ is favoured – a potential explanation for upregulation of p53 seen previously (65). When MAT2A or PRMT5 were inhibited pharmacologically, Kalev et al. observed an increase in R-loop nuclear signals, micronuclei and the DNA damage marker γH2AX (74). The formation of R-loops and consequent DNA damage (and vice-versa) was attributed to irregular splicing arising from lack of PRMT5 activity. Furthermore, this study showed an increase in DIs located in the key DNA damage repair regulator *ATM*, and FA pathway transcripts *FANCL, FANCA* and *FANCD2*, and an associated reduction in protein levels. FA pathway proteins and ATM facilitate HR upon DNA damage (75).

## 4 The function of MAT2A

MAT2A was identified as a top synthetically-lethal hit in three independent shRNA screens in *MTAP*-negative cell lines (18–20). The methionine adenosyltransferase (MAT) enzymes are a family of three proteins that are involved in the synthesis of the molecule SAM (76). The MAT2A substrates methionine and ATP are processed to produce SAM via an adenosine intermediate (77). SAM can then be utilised by methyltransferases, such as PRMT5, for downstream methylation processes. The enhanced expression and activity of MAT2A results in an elevated production of SAM and has been associated with tumour progression in liver cancer (78), hepatocellular carcinoma (HCC) (79, 80) and colorectal cancer (81, 82). Therefore, targeting MAT2A as a possible strategy for treating cancers (especially in *MTAP*-negative cancers) may reduce tumour growth.

## 5 Pharmacological PRMT5/MAT2A inhibition and selectivity for MTAP-deficient cells

A limiting factor to targeting PRMT5 directly is its important role in normal tissue function, but a synthetically lethal interaction with an *MTAP*-negative background should, in principle, provide a suitable therapeutic window. However, since MTA and SAM bind competitively to the substrate binding pocket of PRMT5 (19)​, MTA accumulation downstream of MTAP loss is a ‘double-edged sword’ in the context of PRMT5 inhibition. A SAM-uncompetitive pharmacological inhibitor of PRMT5 (EPZ015666/GSK3326595) was shown to be an effective anti-proliferative agent in mantle cell lymphoma (MCL) models with overexpression of PRMT5 (83). However, EPZ015666 showed no substantial antiproliferative effect in endogenous and engineered *MTAP*-negative cell lines (18) due to increased levels of MTA outcompeting SAM binding; PRMT5 has a lower affinity for SAM than MTA (19, 25, 84). Additional compounds found to bind in a SAM/MTA-competitive manner, including LLY-283 (85) and JNJ-64619178 (86, 87), are also less effective in conditions of elevated MTA levels. Subsequently compounds have recently been produced that interact with PRMT5 when bound to MTA, and which selectively target *MTAP*-negative cancer cells to varying degrees or elicit a synergistic effect in a *MTAP*-negative background (88, 89). Also, interaction between PRMT5 and SAPs can be targeted by BRD0639 and blocking this interaction reduced PRMT5 function and perturbed cellular growth in *MTAP*-negative cell lines *in vitro* (90). Several PRMT5 inhibitors are now in early-stage clinical trials for different types of cancers (**Table 2**).

**Table 2:** Protein arginine methyltransferase 5 inhibitors.

Drug Name	Structure	Clinical trial identifier (if applicable)	Clinical trial stage (if applicable)	Cancer type in trial (if applicable)	Citation
EPZ015666/ GSK3326595	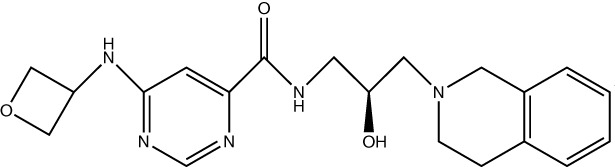	NCT04676516	Phase 2	Early-stage breast cancer	(83)
LLY-283	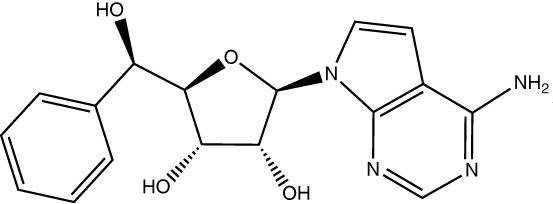				(85)
JNJ-64619178	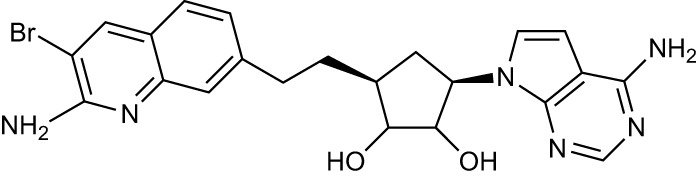	NCT03573310	Phase 1	Neoplasms, Solid tumour (adult), Non-Hodgkin Lymphoma, Myelodysplastic Syndromes	(86, 87)
BRD0639	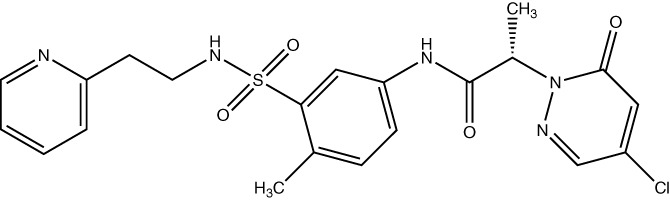				(90)
AMG193	Not published	NCT05094336	Phase 1/2	MTAP-null solid tumours	No publications
TNG908	Not published	NCT05275478	Phase 1/2	MTAP-null solid tumours	No publications
SCR-6920	Not published	NCT05528055	Phase 1	Solid tumour, Non-Hodgkin Lymphoma	No publications
PRT543	Not published	NCT03886831	Phase 1	Solid tumours/ lymphomas, Haematological malignancies	No publications
PRT811	Not published	NCT04089449	Phase 1	Advanced solid tumour, Recurrent Glioma	No publications
MRTX1719	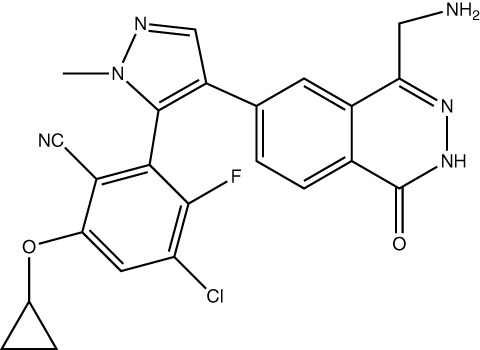	NCT05245500	Phase 1/2	Mesothelioma, Non-small cell lung cancer, Malignant peripheral nerve sheath tumour, Solid tumours, Pancreatic adenocarcinoma	(89)

**Figure 4:**
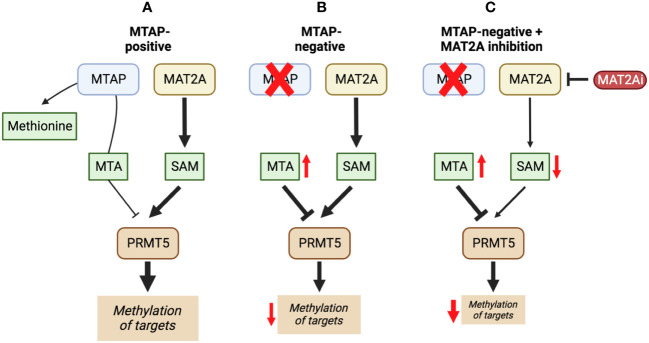
The inhibition of MAT2A exacerbates the reduction in PRMT5 activity present in a MTAP-negative background. Here, we show the changes in PRMT5 activity over different genetic backgrounds [MTAP-positive **(A)** and MTAP-negative **(B)**] and in an MATZA inhibited state within an MTAP-negative background **(C)**. The outcome of these differences is a change in levels of methylation of PRMT5 targets.

Inhibiting MAT2A and reducing SAM levels has been proposed to cause PRMT5 inhibition both by removing its substrate and, in the case of *MTAP*-negative tumours, by providing a greater opportunity for MTA binding. Inhibiting MAT2A will therefore act to reduce protein methylation via PRMT5 (**Figure 4**), in addition to broader metabolism (nucleotide synthesis, glutathione synthesis, etc). Targeting MAT2A rather than PRMT5 directly has shown a greater selectivity for cells with an *MTAP*-negative background (19). As the MAT2A paralog MAT1A is the primary SAM producer in hepatic tissues there is also a lower risk of liver toxicity with selective inhibition of MAT2A (76). The first inhibitors of MAT2A (**Table 3**) were substrate-competitive molecules adapted from the structure of methionine - cycloleucine (91) and aminobicyclohexanecarboxylic acid (92). However, these analogues did not possess the potency and binding specificity for an effective and accurate therapy. The development of small molecules called FIDAS (fluorinated N,N-dialkylaminostilbene) agents showed an improved potency down to low nanomolar concentrations (93, 94). However, the compounds did not show high selectivity for MAT2A at higher drug dosages *in vitro* (94). A non-substrate competitive inhibitor, PF-9366, showed promise with a higher potency for MAT2A; this molecule competitively binds to an allosteric site which mediates interactions with the binding partner MAT2B (95). MAT2B has been suggested to regulate MAT2A in low methionine or high methionine conditions by respectively activating or inhibiting MAT2A activity (95). Other data have suggested that the presence of MAT2B does not affect MAT2A activity but does improve MAT2A stability and longevity in low substrate concentrations (98). Therefore, these data suggest that the capacity of MAT2A inhibition via PF-9366 may be dependent on MAT2B levels or methionine/ATP availability. Extended PF-9366 and cycloleucine treatment led to adaptation in cultured cell lines resulting in an upregulation of MAT2A expression, indicating possible resistance mechanisms (95).

**Table 3:** Methionine adenosyltransferase II alpha inhibitors.

Drug Name	Structure	Clinical trial identifier (if applicable)	Clinical trial stage (if applicable)	Cancer type in trial (if applicable)	Citation
Cycloleucine	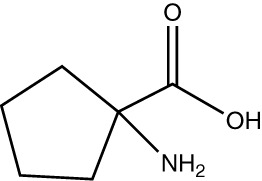				(91)
Aminobicyclo-hexane-carboxylic acid	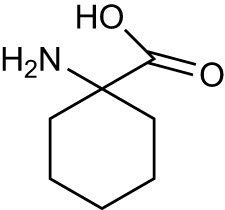				(92)
FIDAS agents (generic structure) - X, Y and Z are variable groups	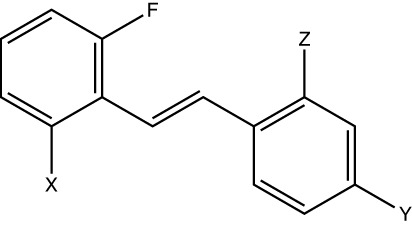				(93, 94)
PF-9366	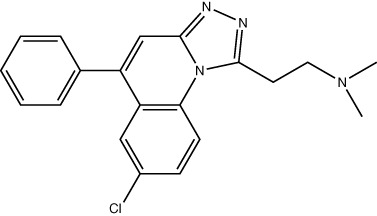				(95)
AGI-25696	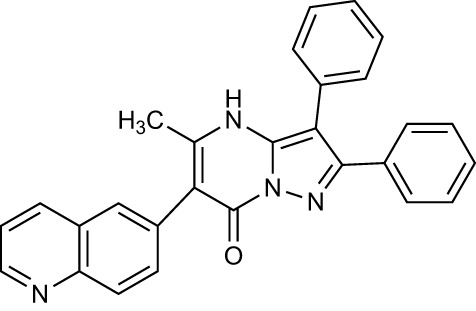				(96)
AG-270 (S095033)	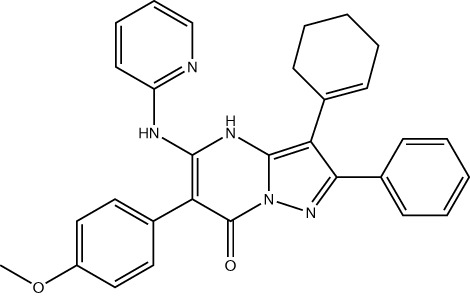	NCT03435250, NCT05312372	Phase 1, Phase 1/2	Advanced solid tumours, Lymphoma, Oesophageal squamous cell carcinoma	(74)
Compound 28	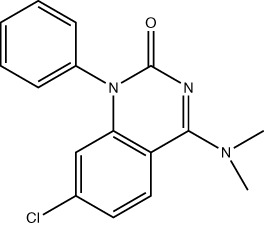				(97)
IDE397	Not published	NCT04794699	Phase 1	Solid tumour	No publications

A series of further non-substrate competitive inhibitors was developed, including an orally bioavailable *in vivo* candidate molecule AGI-25696 (96). AGI-25696 was a poor candidate for human treatment due to high plasma protein binding and consequent low tissue uptake. By masking polarity internally and reducing the hydrogen donors of the molecule, the absorption was improved, and potency maintained (96). The final compound produced, AG-270, has been shown to be effective in reducing proliferation both within *in vitro* cell lines and *in vivo* xenograft models (74). AG-270 is being investigated in a *MTAP*-negative solid tumour and lymphoma phase 1 clinical trial (NCT03435250) and a phase 1/2 clinical trial in advanced and metastatic oesophageal squamous cell carcinoma (ESCC) (NCT05312372). A similar study used a fragment approach to design new MAT2A inhibitors that show a high potency and functionality *in vivo* (97). The study resulted in a drug called compound 28, showed comparable features to AG-270 in that both are orally bioavailable and bind to the MAT2B allosteric region of MAT2A. Both AG-270 (74) and compound 28 (97) showed high potency *in vitro*; reducing proliferation and SDMA markers in a HCT116 *MTAP*-negative background. In a xenograft study of HCT116 *MTAP*-negative tumours AG-270 resulted in 75% growth inhibition (96), while treatment with compound 28 led to complete tumour stasis (97). Another small molecule MAT2A inhibitor by IDEAYA (IDE397) is also in phase 1 clinical trials for *MTAP*-negative solid-tumours (NCT04794699).

PRMT5 clinical trials for PRT543 (NCT03886831) and GSK3326595 (NCT04676516) have been completed and have reported disappointing clinical responses to the monotherapy (99, 100). Just one of the baseline 49 patients achieved complete remission after treatment with PRT543 (101) and GSK has discontinued the trial with GSK3326595 (100). Even though these studies have not reported results selectively in an *MTAP*-negative tumour background, other clinical evidence is emerging that MTAP-negativity does not predict intra tumoral MTA accumulation as seen in model systems (102). This suggests that despite substantive pre-clinical evidence, this genomic biomarker may not sufficiently predict response to MAT2A/PRMT5 inhibition sensitivity in the clinic.

## 6 Determinants of response to MAT2A/PRMT5 targeting beyond *MTAP* status as a route to effective treatment

The selective sensitivity of *MTAP*-negative cancer to MAT2A or PRMT5 inhibition is argued to result from abnormally high levels of MTA. While high MTA levels have been demonstrated extensively in *MTAP*-negative cancer cells *in vitro* (18–20), lower than expected levels of MTA have been observed *in vivo* (102). Barekatain et al. conducted metabolomic analysis of 17 primary glioblastoma multiforme (GBM) tumours, xenograft tumours derived from a series of GBM lines and a 50 GBM tumour metabolomic dataset, which overall showed no significant difference in MTA levels between *MTAP*-negative and *MTAP*-positive primary tumours (102). Consequently, there was not the expected PRMT5 inhibition in primary *MTAP*-negative tumours (shown by a lack of significant reduction in SDMA markers). Barekatain et al. showed evidence that MTA produced from the *MTAP*-negative tumour is being processed by the intratumour, *MTAP*-positive stromal cells. A noticeable finding highlighted that *in vivo* xenografts may not accurately model endogenous tumour response, as xenografts are less populated by stromal cells when comparing their histology to primary GBM tumour tissue (102). These challenges emphasise the importance of reproducible model systems that result in more “patient-like” models. Overall, this study suggests caution in the use of *MTAP* status as the sole predictive biomarker for identifying patients to receive MAT2A/PRMT5 inhibitor therapy.

In general, metabolite levels are likely to differ between cultured cells *in vitro* and patient tumours *in vivo*, not least because the composition of common culture media and levels of oxygenation do not recapitulate the physiological environment where tumours form in the body (103). Furthermore, tissue lineage can also influence the metabolic phenotype of a tumour, even when they share the same oncogenic driver mutations (104). For example, it has been shown that the accumulation of MTA resulting from a homozygous *MTAP* deletion was only reproducible between cell lines when grown in a complete nutrient culture medium, and not when cultured in methionine- or cysteine-depleted media (105). Each cell line in this study demonstrated distinct metabolic profiles upon amino acid restriction, which were clustered more by tissue type than *MTAP* status. These observations also imply that nutrient supply, including dietary methionine or cysteine, could add to patient-to-patient variability in response to therapy targeting the MAT2A/PRMT5/MTAP axis and that combining MAT2A/PRMT5 inhibition with nutrient depletion could result in enhanced responses. Reducing the level of methionine in the body, either enzymatically (106) or through dietary restriction (107), has been shown to reduce tumour volume or increase life span in mice, respectively. Methionine depletion has also been shown to be tolerable with no clinical toxicity in patients (108). When polyamine synthesis is increased in *MTAP*-deleted cells, it can cause reactive oxygen species (ROS) to form and lead to cell death by ferroptosis through lipid oxidation. This effect is amplified by a reduction in cysteine, which impairs the transsulfuration pathway that normally helps to resolve lipid oxidation and reduces the production of glutathione (109).

Given the fundamental importance of SAM levels or arginine methylation in normal tissue function and the systemic impact of MAT2A/PRMT5 inhibitors to lower these, it is important to consider how the combination of such agents with standard-of-care therapies can be rationally designed to benefit from synergy, reduce dosage and alleviate on-target toxicity. In particular, difficult-to-treat cancers could benefit from combination approaches with MAT2A/PRMT5 inhibitors alongside compounds that target the DNA damage response (110).

As described above, PRMT5 activity results in alternative splicing of the transcripts of key DNA damage repair proteins, such as ATM and FA family members, which facilitate repair of double strand DNA breaks, e.g. induced by inter-strand crosslinking. The combination of PRMT5 inhibitors and interstrand crosslinking agents induced an increase in unrepaired inter-strand crosslinks and lead to greater genomic instability (48), implying that MAT2A or PRMT5 inhibitors create a deficiency in the HR DNA repair pathway. Tumours with a defective HR pathway, such as *BRCA2*-negative breast and ovarian cancer, become dependent on PARP1-mediated repair pathways (111). This finding suggests that MAT2A/PRMT5 inhibition may act synergistically in combination with PARP inhibitors, as has been reported in one study of AML (73). PARP inhibitors have also shown significant synergy with type I PRMT inhibitors in NSCLC and in ovarian cancer, where type I PRMT inhibition re-sensitised resistant PEO4 ovarian cancer cells to PARP inhibitor treatment *in vitro* (112). Combination therapy using well characterised chemotherapies alongside PRMT5 inhibitors has shown promising results by targeting both PRMT5 inhibitor sensitive and resistant cells simultaneously. In one study, PRMT5 inhibition resistance was found to be associated with the upregulation of the microtubule regulator, stathmin 2 (STMN2) (113). Upregulation of STMN2 was found to be essential for resistance, and was also responsible for sensitivity to taxanes, such as paclitaxel (113). A combination of MAT2A inhibitors and taxanes was also shown to produce synergy when treating engineered (HCT116) and intrinsically (KP4) *MTAP*-negative cell lines (74).

Pharmacological inhibition of PRMT5 has been suggested to combine effectively with anti-PD1 immune checkpoint therapy (ICT) drugs in different types of cancers. In murine melanoma cell lines, PRMT5 was shown to methylate IFN-γ-inducible protein 204 (IFI16/IFI204) and negatively regulate *NLRC5* transcription (49). Pharmacological inhibition or shRNA knockdown of PRMT5 increased production of type-I interferons by inhibiting IFI16 (IFI204) and increased NLRC5-promotion of major histocompatibility complex (MHC) I antigen presentation genes (49). PRMT5 inhibition was later reported to induce lymphocyte infiltration and MHC II expression in mouse liver HCC tumours, which was demonstrated by an increase in CD45.1, CD4 and CD8 staining in fixed liver tumours and up-regulation of H2-Ab1, Cd74 and MHC II transactivator Ciita at the mRNA level (45). The combination of a PRMT5 inhibitor and anti-PD1 therapy produced a significant reduction in tumour volume and an increase in CD4+ and CD8+ T cell infiltration compared to either therapy alone *in vivo* (45). In contrast, PRMT5 inhibition has also been reported to promote PD-L1 expression in lung cancer and ultimately disrupt antitumour immunity by increasing the marker for immune inhibition (114). While combining PRMT5 inhibition with PD-L1 therapy has potential benefits, it has been reported that the MTA-rich environment in *MTAP*-negative cancer cells stimulate the immunosuppressive (M2) state in macrophages through activation of the adenosine A_2B_ receptor (115), thus inhibiting immune invasion. SAM and MTA secreted by tumours can reduce global chromatin accessibility in T cells, leading to dysfunction that may contribute to a poor anti-tumour immune response (116). As such, investigating the impact of MAT2A inhibitors on the function of tumour-associated T cells may provide additional insight into how the efficacy of immune-checkpoint inhibitors could be improved.

## 7 Conclusions

Deletion of *MTAP* is frequently observed in a wide variety of cancers due to its proximity to the key tumour suppressor gene *CDKN2A*. The codeletion of *MTAP* provides a selectable marker for the identification of cancer patients who might benefit from targeting of methionine metabolism and/or protein methylation due to accumulation of the metabolite MTA. Here we have reviewed current pharmacological methods of targeting SAM production via MAT2A inhibition and direct PRMT5 inhibition (both of which reduce PRMT5-specific methylation reactions) and reported evidence for and against the selectivity of these treatments for *MTAP*-negative tumours. Future generations of drugs that target PRMT5 activity in an *MTAP*-negative background must focus on maximising their selectivity for inhibiting PRMT5 specifically within the cancer cell (i.e. when PRMT5 is bound to MTA). Moving forward, to best understand the population of patients that will benefit from therapeutic treatment with MAT2A/PRMT5 inhibitors we require a greater understanding of the molecular rewiring after inhibition. Our current understanding of the downstream effects of PRMT5 inhibition include the impairment of pre-mRNA splicing and DNA damage repair, co-treatment of cancers with agents that target these pathways such as PARP inhibitors and chemotherapy, is potentially synergistic. Such a strategy provides an alternative rationale for the use of MAT2A/PRMT5 inhibitors beyond synthetic lethality with *MTAP* loss and presents additional predictive biomarkers for future clinical development of combination treatments.

## Author contributions

CB: Writing – original draft. CB: Writing – review & editing. IM: Writing – review & editing. HK: Writing – review & editing.
